# Effects of combination approach on harm reduction programs: the Taiwan experience

**DOI:** 10.1186/s12954-016-0112-3

**Published:** 2016-07-04

**Authors:** Ting Lin, Chang-Hsun Chen, Pesus Chou

**Affiliations:** Community Medicine Research Center and Institute of Public Health, National Yang-Ming University, No.155, Section 2, Ni-Long Street, Taipei, 11221 Taiwan; Taiwan AIDS Foundation, No. 410, 8F, Nanjing W. Rd., Tatung Dist., Taipei, 10343 Taiwan; Taiwan Centers for Disease Control, Ministry of Health and Welfare, No.6, Linsen S. Rd., Zhongzheng Dist., Taipei, 10050 Taiwan

**Keywords:** Human immunodeficiency virus, Comprehensive harm reduction programs, Injection drug users, Needle and syringe exchange programs, Opioid substitution therapy, Combination intervention approaches, Forced withdrawal, Relapse

## Abstract

**Background:**

In 2003, a major epidemic of human immunodeficiency virus emerged among injection drug users in Taiwan. In response to the twin epidemics of HIV and intravenous drug addiction, the government implemented comprehensive harm reduction programs beginning in 2005. Collected data from relevant agencies were used to explore the impact of the harm reduction programs on HIV and illicit drug use.

**Methods:**

This study divided 2002–2015 into three intervention phases and used the surveillance data and statistics on the HIV epidemic, drug abuse, and the intervention from relevant agencies to explore the correlations between different variables in different intervention periods and the combination effects of interventions on the HIV epidemic.

**Results:**

In the pre-intervention phase, the growth of the HIV epidemic followed the rapidly increasing number of heroin users, reaching a peak in 2005. After the initiation of harm reduction programs, the HIV epidemic ceased growing, even rapidly declining with the expansion of needle and syringe exchange programs and opioid substitution therapy; however, the number of heroin users remained high. When the implementation of the needle and syringe exchange programs and the opioid substitution therapy program reached the plateau level in the consolidation phase, the number of heroin users also decreased rapidly. The combination effects of the harm reduction programs in this period also pushed levels of HIV infection below those before this outbreak.

**Conclusions:**

The HIV epidemic among injection drug users incorporates the dual problems of drug addiction and needle-sharing behaviors, so the use of a single intervention will not resolve all of the problems. Facing a severe HIV epidemic among injection drug users, quickly scaling up and promoting comprehensive harm reduction programs is a good strategy that can be used to simultaneously reverse the HIV epidemic and to resolve the illicit drug use problems.

More research is needed to find out the reasons behind why there were cases that declined opioid substitution therapy, so that efforts can be undertaken to avoid the epidemic rebounding.

## Background

Globally, injection drug users (IDUs) have been identified in at least 158 countries and territories [1]. IDUs are susceptible to blood-borne infectious diseases, such as human immunodeficiency virus (HIV) infection [2–4]. In 2013, the number of IDUs worldwide was estimated to be around 12.19 million (range 8.48–21.46 million), and among these, about 1.65 million (range 0.92–4.42 million) were estimated to be infected with HIV [5].

Comprehensive harm reduction programs are remarkably effective in controlling HIV among IDUs and are also recommended by the Joint United Nations Program on HIV/AIDS (UNAIDS), the United Nations Office on Drugs and Crime (UNODC), and the World Health Organization (WHO) [6]. Cost-effective interventions, including needle and syringe exchange programs (NSPs), opioid substitution therapy (OST), and expanded access to HIV treatment and care, are supported on public health and human rights grounds [7, 8]; however, at the global level, harm reduction responses related to unsafe injection remain poor [5]. In 2014, 91 countries included harm reduction programs in their national policies, NSPs were available in 90 countries, and OST was available in 80 countries. Moreover, in many countries, the availability of harm reduction services remain at levels below minimum levels recommended by international guidance [5, 9].

It is crucial for regions with rapidly growing epidemics to optimize the effectiveness and coverage of interventions. Each intervention will achieve only modest reductions in HIV transmission alone, and the elimination of HIV transmission necessitates high coverage and combined approaches [10–12]. In 2003, a major HIV epidemic among IDUs emerged in Taiwan. Following the rapid increase in the heroin users, the number of IDUs newly infected with HIV also increased from 80,620 (9.3 %, 40.8 % of all newly reported HIV cases) in 2003 and 2004, respectively, to a peak level of 2420 (71.6 % of all newly reported HIV cases) in 2005.

In response to this HIV epidemic, the government secured a political commitment to implement comprehensive harm reduction programs in 2005. We collected nationwide data from relevant agencies to explore the impact of harm reduction programs on HIV and illicit drug use in Taiwan.

## Methods

The materials used in this research included data from the following agencies: (a) 2002–2015: the annual number of newly reported HIV infection cases and cases among IDUs collected from the national HIV/AIDS reporting system of the Taiwan Centers for Disease Control (TCDC) [13]; (b) 2002–2015: the number of “urine specimens that have a morphine reaction” and the number of heroin users reported by hospitals collected from the Food and Drug Administration [14]; (c) 2003–2014: the percentage of drug delivery methods among drug addicts reported from hospitals collected from the Annual anti-drug report [15]; (d) 2005–2015: the number of NSP sites and the total annual number of needles-syringes distributed collected from the data of harm reduction programs in the TCDC [16]; (e) 2006–2012: the number of OST clinics, the number of cases in OST, and the total annual person-days collected from TCDC [17]; and (e) additional data from 2013 to 2015 collected from the Ministry of Health and Welfare [18].

The frequency distribution of the variables in this study are described in Table 1.

**Table 1:** The frequency distribution of variables from 2002 to 2015

Year	Urine specimens with a morphine reaction^*^ (low estimates of IDUs) (a)	Hospital reported heroin cases^¥^ (b)	The estimates of IDUs (a) + (b)	Newly reported HIV infection cases (c)	HIV incident cases among IDUs (d)	% of (d)/(c)	No. of NSP sites^#^	Total annual needles-syringes distributed	No. of OST clinics^¶^	No. of cases on OST^§^	Total annual person- days
2002	23,385	6233	29,618	767	18	2.3					
2003	27,741	7353	35,094	860	80	9.3					
2004	32,283	11,479	43,762	1521	620	40.8					
2005	39,123	11,466	50,589	3380	2420	71.6	76	12,568			
2006	34,775	11,219	45,994	2918	1839	63.0	729	438,081	21	641	66,287
2007	36,625	17,614	54,235	1930	742	38.4	1194	3,634,414	71	5585	1,225,027
2008	36,362	20,096	56,458	1742	386	22.2	1521	4,066,114	90	12,598	3,071,639
2009	24,516	17,657	42,173	1644	178	10.8	1272	3,097,348	97	11,519	2,904,644
2010	21,505	17,176	38,674	1795	116	6.5	1331	3,586,071	100	11,750	2,964,509
2011	18,501	14,020	32,521	1968	111	5.6	1302	3,497,991	102	11,991	3,107,275
2012	18,668	12,429	31,097	2222	86	3.9	1364	3,557,660	102	11,127	3,113,635
2013	14,541	13,458	27,999	2243	48	2.1	1353	3,259,129	117	10,651	3,567,611
2014	12,666	11,298	23,964	2235	52	2.3	1278	3,674,650	133	9231	2,553,545
2015	14,260	11,697	25,957	2327	82	3.5	1252	3,860,518	162	8789	2,570,744

^*^Heroin in the body will be metabolized into morphine. Specimens came from the drug abuse urine-testing laboratory accredited by the Ministry of Health and welfare, the Food and Drug Administration, county and city health bureau, Ministry of Justice Investigation Bureau, National Police Agency Criminal Investigation Bureau, and the Military Police Command

^¥^These cases do not include OST cases

^#^NSP sites include pharmacy-based NSP sites and vending machines

^¶^OST clinics include the clinics of psychiatric hospitals and the clinics of local health stations

^§^Persons whose attendance rate in OST program was over 70 %

In order to explore the correlation between different variables in different periods, the combination effects of the interventions on the HIV infection were also evaluated. For the figures, time was plotted on the horizontal axis and the frequency distribution of the variables was plotted on the vertical axis. We divided the whole period into three different phases of intervention. The pre-intervention phase is the time before August 2005. In November 2008, the OST program reached the plateau level, so the time after August 2005 to November 2008 was set as the expanding phase. The period of time after November 2008 was set as the consolidation phase.

To compare the correlation of the two continuous variables, we used the Person correlation method to calculate the correlation coefficient (*r*). When calculating the combined effects of NSPs and OST, we used the multiple-regression method. We used SPSS statistical software to analyze the data, and a *p* value ≤0.05 was considered statistically significant.

## Results

### The temporal trend and correlation of illicit drug use and the HIV epidemic among IDUs

In Taiwan, there is no long-term epidemiological studies on the prevalence of opiate use in the general population. Therefore, some data from the Food and Drug Administration was used as indicators to represent the data of IDUs. The first data is the number of “urine specimens that have a morphine reaction.” These specimens originated from many different agencies and institutes whose sources are more comprehensive (Table 1). We used these numbers as low estimate numbers of IDUs, and these numbers plus the numbers of heroin cases reported by the hospitals were used as the estimate numbers of IDUs.

In 2003, a major HIV epidemic among IDUs emerged in Taiwan. Following the rapidly increasing number of heroin users, the number of IDUs newly infected with HIV also increased from 80 (9.3 % of all new HIV cases) to its highest point 2420 (71.6 % of all new HIV cases) in 2005. After the initiation of harm reduction programs, it stopped growing and declined quickly. Between 2005 and 2013, the number of newly infected HIV cases among IDUs decreased from 2420 (71.6 % of all new HIV cases) to 48 (2.1 % of all new HIV cases); however, it increased slightly to 82 (3.5 % of all new HIV cases) in 2015.

During the early stages of intervention, the drug addiction epidemic remained at a plateau level for a few years and did not decline until 2008 (totaling 50,589, 45,994, 54,235, and 56,458 in 2005–2008, respectively). After the implementation of the OST program reached a plateau level in 2008, and the stages of the intervention passed over into the consolidation stage, the drug addiction epidemic also reversed, entering a rapid decline, from 42,173 in 2009 to 23,964 in 2014, even lower than 29,618 in 2002. However, it slightly increased to 25,957 in 2015.

**Fig. 1:**
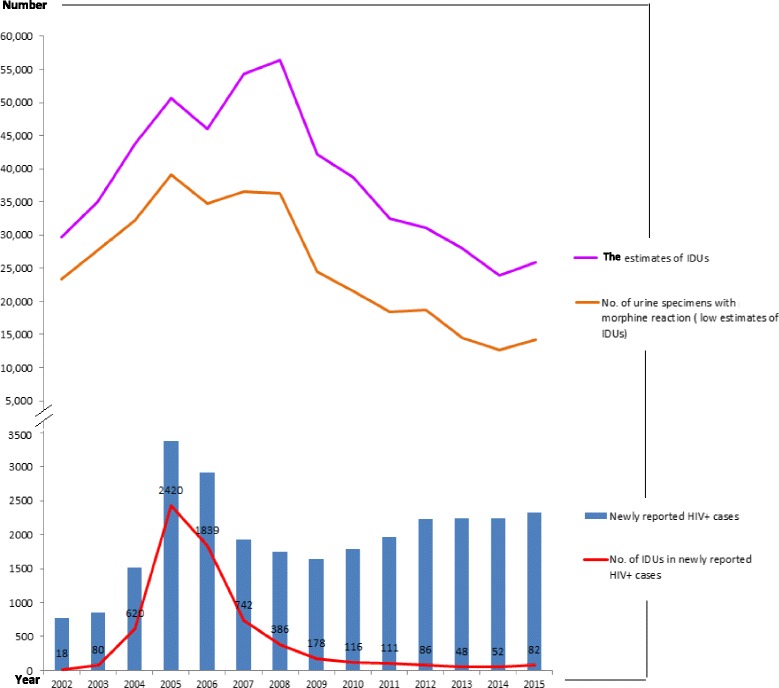
The temporal trend and correlation of illicit drug use and HIV epidemic among IDUs. The relationships were positively correlated, *r* = 0.61, *p* < 0.05, and *r* = 0.72, *p* < 0.01

Speaking generally, we found that illicit drug use had a strong influence on HIV infection among IDUs, and the trend of HIV infection among IDUs followed the trend of both estimated numbers of illicit drug use (compared with the estimates, *r* = 0.61, *p* < 0.05; and compared with the low estimates, *r* = 0.72, *p* < 0.01, Fig. 1).

### The correlation of NSP’s implementation and the trend of HIV infection among IDUs

**Fig. 2:**
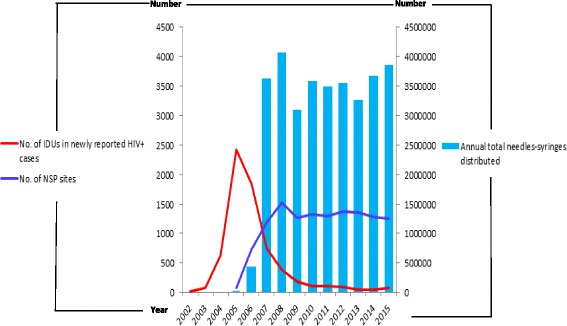
The correlation of NSP’s implementation and the trend of HIV epidemic among IDUs. The relationships were negative correlated, *r* = −0.94, *p* < 0.01, and *r* = −0.94, *p* < 0.01

Following the implementation of pilot programs in four administrative areas, NSPs were introduced in November 2005, and the first clean syringe dispensary was initiated at the same time. At the end of year 2005, there were 76 dispensing sites and 12,568 clean syringes had been dispensed. After evaluating the effectiveness of the pilot plan, the Ministry of Health initiated an expansion of this program nationwide in July 2006, quickly setting up clean needle exchange stations in 23 administrative areas; by the end of 2006, the number of NSP sites had reached 729, and the number of needles and syringes distributed rose up to 438,081 items. NSP coverage reached a plateau in 2007, peaking in 2008, when a total number of 1521 distribution sites had been set up and a total of 4,066,114 needles and syringes had been distributed. The NSP trend then remained on this plateau until 2015, with an average level of about 1300 distribution sites set up per year and issuing about 3.5 million pieces of clean needles and syringes each year. The trend of HIV infection among IDUs stopped growing after the harm reduction programs began and declined subsequent to NSP growth. There was a negative correlation between the trends of HIV infection among IDUs and NSP implementation (compared with the total annual number of needles and syringes distributed, *r* = −0.94, *p* < 0.01; and compared with the number of NSP sites, *r* = −0.94, *p* < 0.01, Fig. 2).

In 2005–2008, when the NSP implementation grew rapidly, the number of heroin addicts remained at a plateau level. This seemed to show that NSPs solved the problem of sharing behaviors, which quickly reduced HIV infection rates. However, fundamentally, NSPs cannot solve addiction problems, and many people continue to use drugs.

### The correlation of OST implementation and the trend of heroin addiction epidemic

The OST program began in February 2006 with the first heroin addict being admitted to the Taoyuan Psychiatric Hospital to receive methadone maintenance treatment. This later expanded to the whole country in August 2006. At the end of 2006, 21 methadone clinics had been established, with 641 individuals regularly taking medicine from them; the total annual person-days reached 66,287. The implementation of the OST program reached a plateau level in 2008 (90 clinics, 12,598 persons regularly receiving medication, and the total annual person-days was 3,071,639). OST coverage was maintained at a plateau level for years, until 2013 (with an average level of about over 100 clinics set up, about 11,000 persons regularly taking the medicine, and about 3 million total annual person-days). Later, in the years 2014 and 2015, the coverage of the OST program was slightly reduced; however, it was still maintained to a considerable degree.

**Fig. 3:**
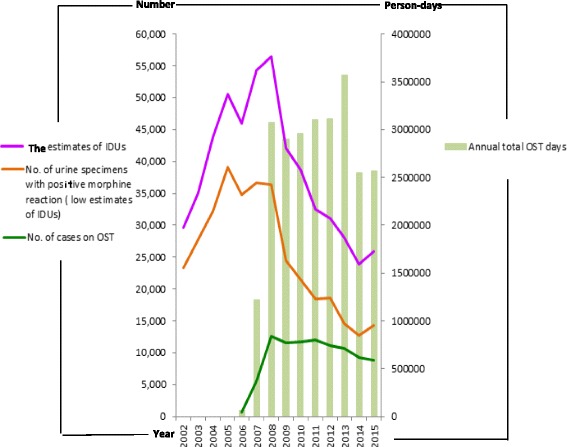
The correlation of the OST program implementation and the trend of the heroin addiction epidemic. They were negatively correlated, *r* = −0.43, *p* = 0.11 and *r* = −0.60, *p* < 0.05

The number of heroin addicts increased rapidly from 2003 reaching the plateau in 2005. Although harm reduction programs began in August 2005, the OST program began later than the NSP program and also reached a plateau later than the NSP program. Therefore, the drug addiction epidemic was maintained at a plateau level and did not decline until 2008. Following the implementation of the OST program reaching a plateau level in 2008, the number of drug addicts also reversed, rapidly declining from 42,173 in 2009 to 23,964 in 2014. In 2015, following the coverage of the OST program being slightly reduced, the number of heroin users also increased to 25,957. There were negative correlations between the implementation of the OST program and the trends of the heroin addiction epidemic (negative correlation between the annual total person-days with both of the two estimated numbers of heroin users, *r* = −0.43, *p* = 0.11, and *r* = −0.60, *p* < 0.05, respectively (Fig. 3)).

The correlation coefficient of the OST program and the low estimates of IDUs (the number of urine specimens with a morphine reaction could be referred to as the number of heroin users not linked to medical care) was *r* = −0.60, *p* < 0.05. After taking into account the number of heroin users that are linked to medical care, the correlation coefficient decreased to *r* = −0.43, *p* = 0.11. Therefore, the implementation of OST program can reduce the number of heroin addicts, especially those who are not linked to medical services.

### Combination effects of harm reduction interventions in different intervention periods

The whole period of 2002–2015 was divided into three different intervention phases. During the pre-intervention phase, there were no harm reduction implementations initiated in this period, apart from educational projects. Illicit drug use and the HIV infection cases among IDUs increased very rapidly. The trend of HIV infection among IDUs peaked in 2005, and after the initiation of harm reduction programs, the trend of HIV infection among IDUs stopped increasing.

During the expansion phase of the harm reduction programs, the HIV epidemic rapidly declined with the expansion of NSPs and the OST program. However, the number of heroin users remaining at a plateau level did not decrease at that time. Although the expansion of the OST program occurred later than that of the NSPs, the OST program still influenced the HIV epidemic. There were negative correlation between the trends of HIV infection among IDUs and OST implementation (compared with the total annual person-days, *r* = −0.92, *p* < 0.01; compared with the number of cases on OST program, *r* = −0.89, *p* < 0.01; and compared with the number of OST clinics, *r* = −0.86, *p* < 0.01).

When the implementation of the NSP and OST programs reached a plateau in the consolidation phase, the OST program reached a high level of effectiveness, and the number of heroin users decreased steeply. The combination effects of harm reduction programs in this period also dropped the number of HIV infection among IDUs eventually to levels from before the beginning of the epidemic.

**Fig. 4:**
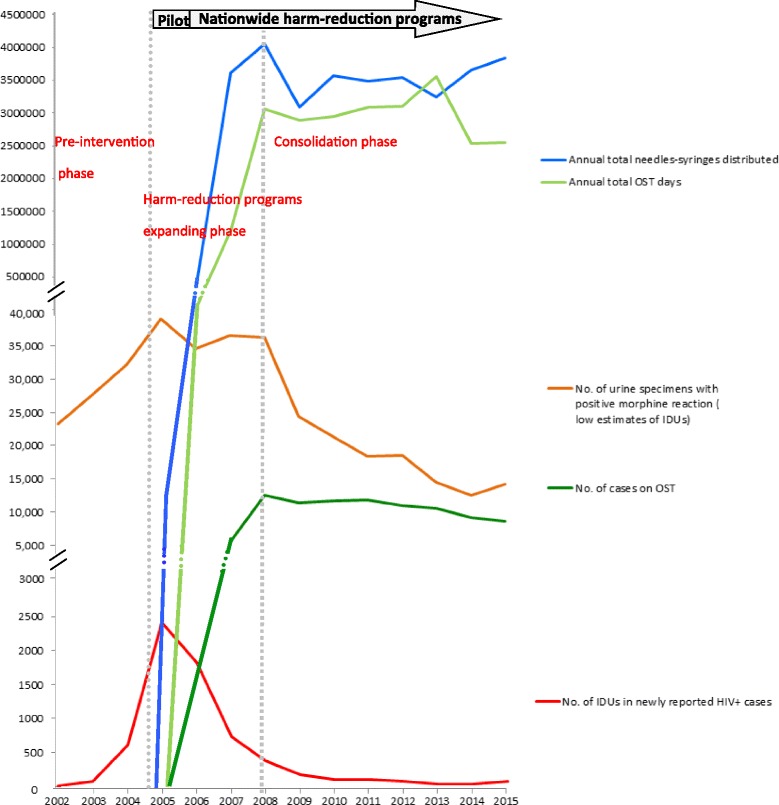
Combination effects of harm reduction interventions in different intervention periods. Each intervention variable of NSPs or OST program independently influenced the HIV epidemic. When the combined effects of NSPs and the OST program were taking into account, the coefficient of determination (*R* ^2^) value of NSPs when controlled of OST (*R* ^2^ = 0.746) and the value of OST program when controlled of NSPs (*R* ^2^ = 0.852), respectively, increased to *R* ^2^ = 0.922

When calculating the combined effects of NSPs and OST, we used the multiple-regression method. Each intervention variable of NSPs or OST program independently influenced the HIV epidemic. When the combined effects of NSPs and the OST program were taken into account, the coefficient of determination (*R*^2^) value of NSPs when controlled for OST (*R*^*2*^ = 0.746) and the value of the OST program when controlled for NSPs (*R*^*2*^ = 0.852) increased to *R*^*2*^ = 0.922; the explained variance of the dependent variable increased by 17.6 and 7.0 %, respectively (Fig. 4).

Suppose the estimate number in our study is an unbiased estimate of IDUs, then we can use the estimate number of IDUs to calculate the coverage level of NSPs and the OST program. During the consolidation phase, the average number of needles obtained by each person in each year was 123 (range 92–149), and the average percentage of OST participants among IDUs was 34.8 % (range 27.3–39.5 %). When compared to other countries, the coverage level was satisfactory.

### The effects of harm reduction interventions on needle-sharing behavior

From the annual anti-drug report, there were some data from hospitals describing the proportion of different patterns of drug use. Even though it did not distinguish opiates with other drugs, the pattern of drug use by injection and needle-sharing behavior was described in these reports. We included it to present the effects of harm reduction interventions on needle-sharing behavior (Table 2).

**Table 2:** The percentage of the pattern of drug use by injection and needle-sharing behavior among drug user reported by hospital

Year	No. of drug user reported by hospitals	Percentage of IDUs among drug user	Percentage of needle-sharing behavior among drug user	Percentage of needle-sharing behavior among IDUs
2003	8283	63.2	15.3	24.2
2004	12,232	63.9	15.0	23.5
2005	12,258	56.3	11.6	20.6
2006	11,967	58.9	10.7	18.2
2007	18,776	65.3	10.1	18.3
2008	21,574	67.3	5.2	7.7
2009	19,125	69.7	7.2	10.3
2010	18,792	70.6	6.1	8.6
2011	17,076	68.6	3.4	5.0
2012	18,562	57.2	3.0	5.2
2013	19,535	58.8	2.6	4.4
2014	17,896	50.9	2.6	5.1

**Fig. 5:**
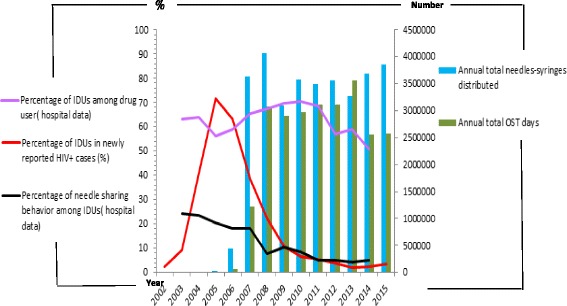
The effects of harm reduction interventions on needle-sharing behavior. They were negatively correlated, *r* = −0.74, *p* < 0.01 and *r* = −0.90, *p* < 0.01

From the data reported by hospitals in Fig. 5, we found that the percentage of IDUs among drug user fluctuated up and down during 2003 to 2014, but the trend of needle-sharing behavior among IDUs declined gradually following the initiation and expansion of the harm reduction programs. Needle-sharing behavior declined steeply when the implementation of the harm reduction programs reached the high level seen in 2008. The trend of sharing behavior was also consistent with the trend of the HIV epidemic. The implementation of NSPs and OST program were negatively correlated with the trend of sharing behavior (compared with the annual total needles-syringes distributed, *r* = −0.74, *p* < 0.01; and compared with the annual total person-days, *r* = −0.90, *p* < 0.01).

## Discussion

In this study, we found that:

1. The illicit drugs, particularly heroin, have a strong impact on the HIV epidemic among IDUs.
2. The NSPs can contribute to the control of the HIV epidemic. However, NSPs cannot solve the drug addiction problem among IDUs.
3. The implementation of OST programs can reduce the number of heroin addicts, especially those who are not linked to medical services.
4. The popularity of the harm reduction service sites improved the accessibility of services and involved more of the target population to participate in the programs.
5. A combination approach of interventions can increase the synergistic effects of the individual programs on HIV epidemic.
6. The implementation of NSPs and OST programs can reduce the proportion of IDUs engaging in needle-sharing behaviors.

Some other key factors for the success of harm reduction programs in Taiwan can also be analyzed as followed:

1. To confront the epidemics of illicit drug use and HIV among IDUs, early alerts and rapid responses are crucial.
2. The rapid expansion from a pilot plan limited to four administrative areas to nationwide harm reduction programs within a year quickly amplified the coverage levels of the interventions.
3. Through communication, a consensus was reached among law enforcement agencies and public heath sectors to support the implementation of the programs and to create good cross-sectoral cooperation at the level of both central and local government.
4. Consistent cooperation with international partners and studying and learning fully from the successful experience of other countries shortened the period of trial and error.
5. Through the engagement of stakeholders, critical information, such as feedback from drug users and peer volunteers of non-governmental organizations (NGOs), can be used to amend harm reduction programs and to tailor the programs to meet the needs of the target population [19].

Even though the harm reduction programs were successfully implemented in Taiwan, a new problem, IDUs dropping out of the OST program has emerged. In Taiwan, the OST program has only been implemented in communities and not in the correctional system. When people who are receiving methadone maintenance treatment for opioid dependence become incarcerated, methadone treatment is discontinued abruptly. Non-governmental organization Taiwan AIDS Foundation (TAF) regularly engages in health education in correctional facilities. According to peer volunteers of TAF, because OST is ended abruptly once a person is incarcerated, the withdrawal symptoms that can last 2–3 weeks or more often cause the incarcerated to hesitate to return to the treatment system. This discontinuation situation, which has also been observed in other countries, can induce uncomfortable symptoms of withdrawal and cause prisoners to be susceptible to relapse and overdose on release [20, 21]. We found that the coverage of OST is slightly reduced from 2013 to 2015 (even though sites providing medication increased from 117 to 162, the number of regular cases in OST have declined from 10,651 to 8789, and the total annual person-days have also declined from 3,567,611 to 2,570,744). At the same time, as the coverage of the OST program declined, the number of heroin users fluctuated down and up from 27,999 to 23,964 to 25,957, and the number of newly reported HIV cases among IDUs also increased from 48 to 82. This situation acts as an early warning about challenges to the long-term success of the current programs. More research is needed to find out the reasons behind why there were cases that declined opioid substitution therapy, so that efforts can be undertaken to avoid the epidemic rebounding.

Our study has some limitations. Core intervention components should include access to antiretroviral therapy, but antiretroviral therapy has been available free of cost in Taiwan since 1997 even in prisons, so data on the access to antiretroviral therapy was not included in this study. Information, education, and communication (IEC) are also very important; feedback from drug users and peer volunteers of NGOs can be used to amend the harm reduction programs and to tailor the programs to meet the needs of the target population [19]. However, most of the education programs either have been implemented before the harm reduction programs or have been integrated into OST or NSPs programs, and there is a lack of quantitative data, so it was not regarded as a separate factor in this study. Even so, with these limitations, there has been a study using an individual-level analysis of the amnesty cohort in Taiwan. They found that attendance at methadone clinics was associated with a significantly lower HIV incidence, and frequent users of needle/syringe program services had lower HIV incidence [22]. These findings also support our study. Without cross-country comparisons, the inferences of the research results must also be restricted. However, country-level comparisons and modeling projection studies in western countries have shown that the extent of coverage of OST and NSPs is associated with the incidence and prevalence of HIV among IDUs. The experiences of scaled-up harm reduction responses in Malaysia have provided an example of effective practices that can be used in Asia. All these evidence-based studies support our findings [23–25]. From a global point of view, the level of harm reduction responses related to unsafe injection remains poor, and program implementation remains at levels below the international minimum guidelines in many countries [5]. We have sufficient knowledge on which actions are effective, but the problem is implementing these actions well and to the appropriate scale. In this study, nationwide data from various surveillance systems that covered the different phases of the interventions were collected, and the mechanism of actions that could provide a complete picture of harm reduction implementation in Taiwan was analyzed. Taiwan’s experience can be used as a reference by countries suffering from HIV epidemics among IDUs, so that they may more effectively assess and implement their national intervention programs.

## Conclusions

The implementation of these government-initiated comprehensive harm reduction programs has curtailed the HIV epidemic among IDUs in Taiwan. Our findings suggest that countries suffering from HIV epidemics among IDUs should offer comprehensive harm reduction programs and should fully scale up such implementations for their population.

More research is needed to find out the reasons behind why there were cases that declined opioid substitution therapy, so that efforts can be undertaken to avoid the epidemic rebounding.

## Abbreviations

HIV, human immunodeficiency virus; IDUs, injection drug users; IEC, information, education, and communication; NGOs, non-governmental organizations; NSPs, needle and syringe exchange programs; OST, opioid substitution therapy; TAF, Taiwan AIDS Foundation; TCDC, Taiwan Centers for Diseases Control; UNAIDS, Joint United Nations Program on HIV/AIDS; UNODC, United Nations Office on Drugs and Crime; WHO, World Health Organization
